# Supplementary material for: Break-induced replication promotes formation of lethal joint molecules dissolved by Srs2
Source: Nat Commun. 2017 Nov 27;8:1790. doi: 10.1038/s41467-017-01987-2 (PMC5702615; doi:10.1038/s41467-017-01987-2)
Supplement: Supplementary file 1 — Supplementary Information [file 41467_2017_1987_MOESM1_ESM.pdf]

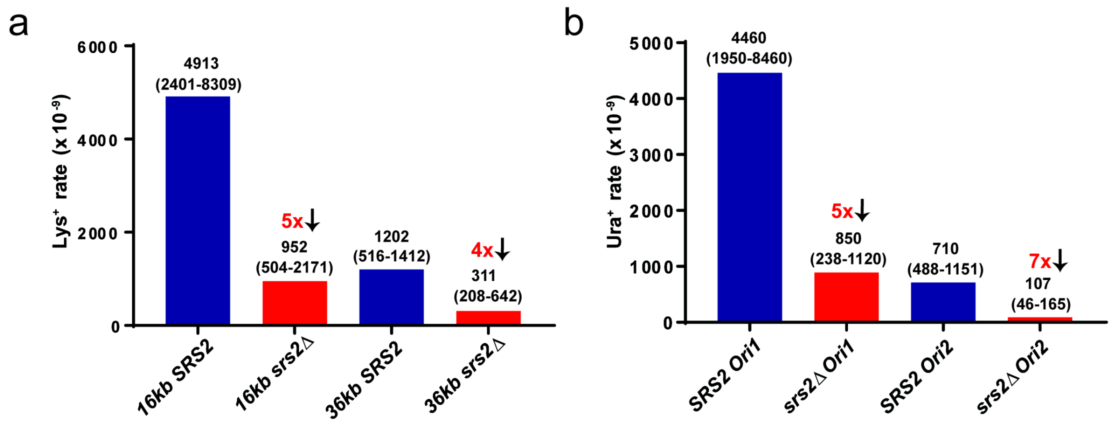

Supplementary Figure 1. The effects of *srs2Δ* on frameshift and base-substitution mutagenesis. (a) Analysis of frameshift mutagenesis in *SRS2* and *srs2Δ* using *lys2::A$_4$* reporter [34] inserted in the donor chromosome at positions 16kb and 36kb from the DSB. *SRS2* (wild type) data are from[34]. (b) Analysis of base-substitution mutagenesis using *ura3-29* reporter inserted in two orientations (Ori1 and Ori2; [10]) at a position 36kb from DSB in *SRS2* and *srs2Δ*. Mutation rates are reported as the median value with range (in parenthesis) calculated based on ≥ 4 individual experiments. Downward arrows represent statistically significant decrease as compared to *SRS2* ($P<0.05$ using non-parametric Mann-Whitney test).

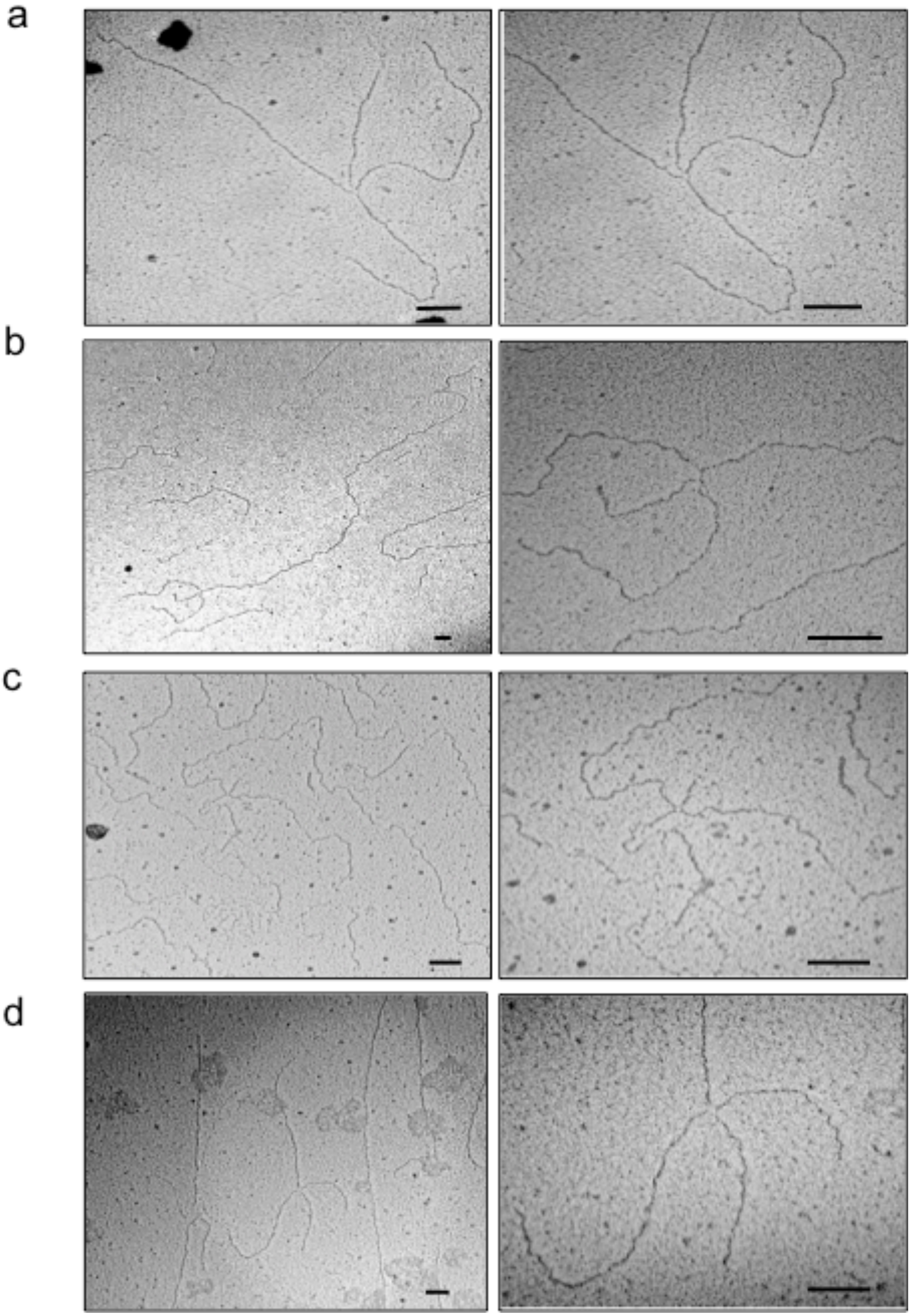

Supplementary Figure 2. Representative EM images showing 4-way junctions in *srs2Δ*. (a-d) Entire field images including 4-way junctions are shown (left) and enlarged views are shown (right). Scale bars correspond to 100nm.

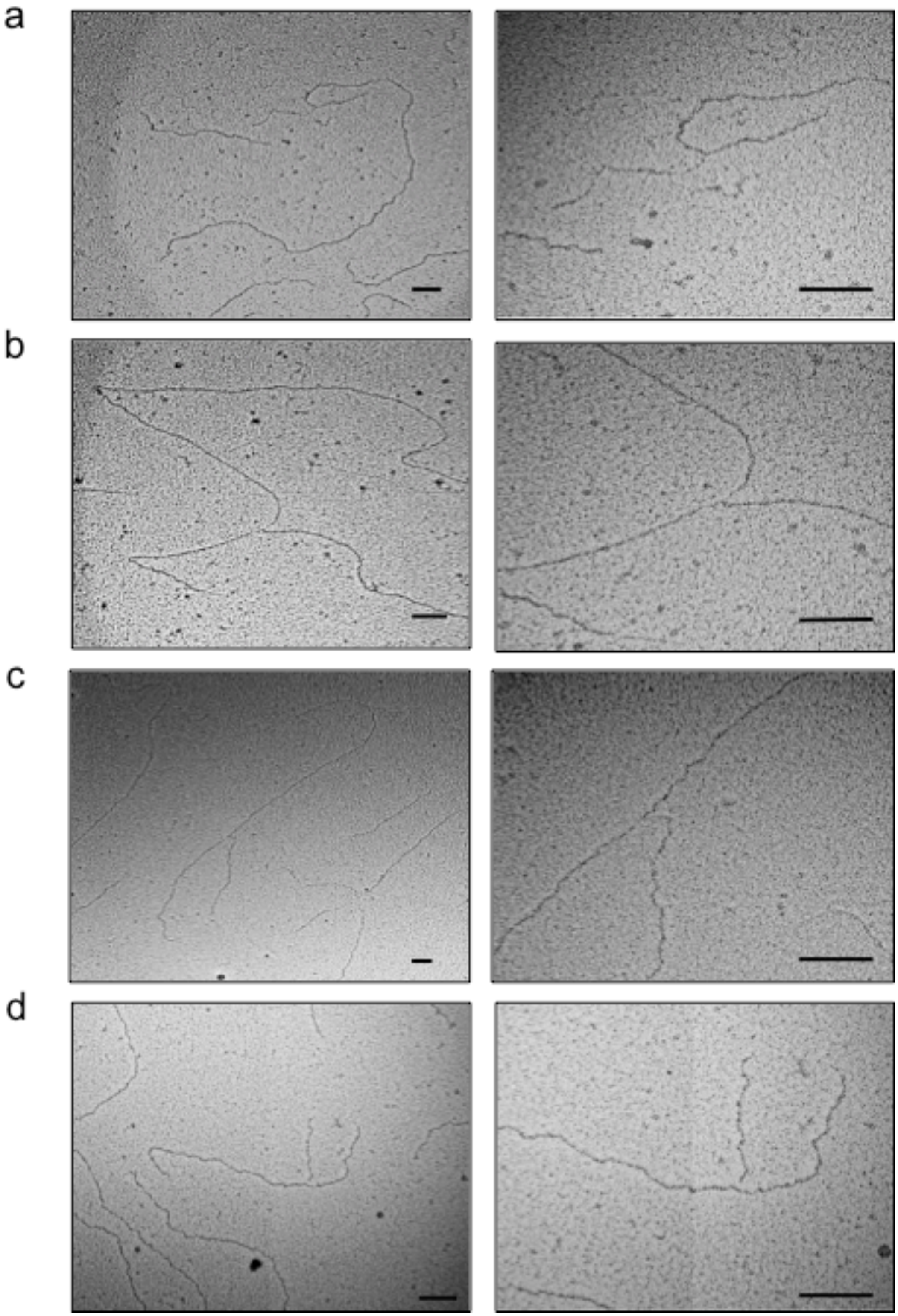

Supplementary Figure 3. Representative EM images showing 3-way junctions in *srs2Δ*. (a-d) Entire field images including 3-way junctions are shown (left) and enlarged views are shown (right). Scale bars correspond to 100nm.

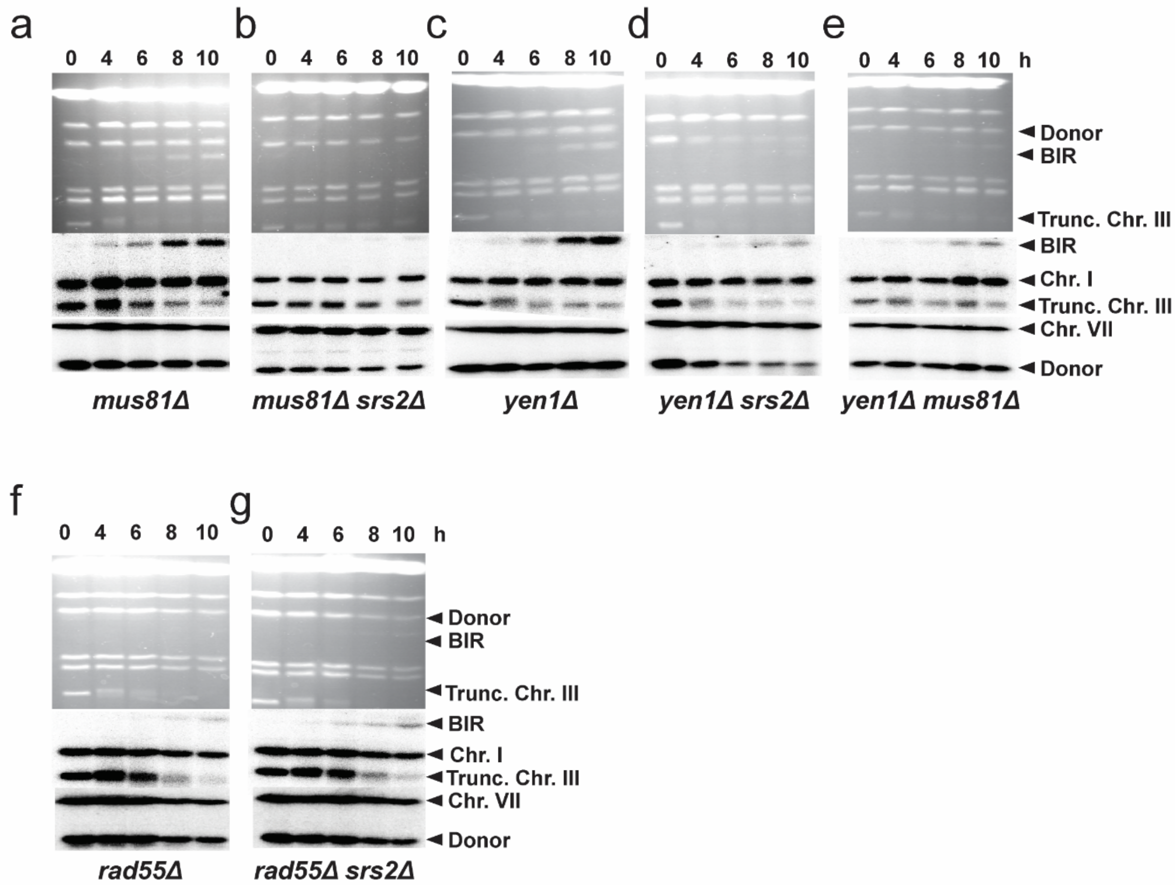

Supplementary Figure 4. Physical analysis of BIR using CHEF gel electrophoresis in the following mutants: (a) *mus81Δ*, (b) *mus81Δ srs2Δ*, (c) *yen1Δ*, (d) *yen1Δ srs2Δ*, (e) *yen1Δ mus81Δ*, (f) *rad55Δ*, and (g) *rad55Δ srs2Δ*.

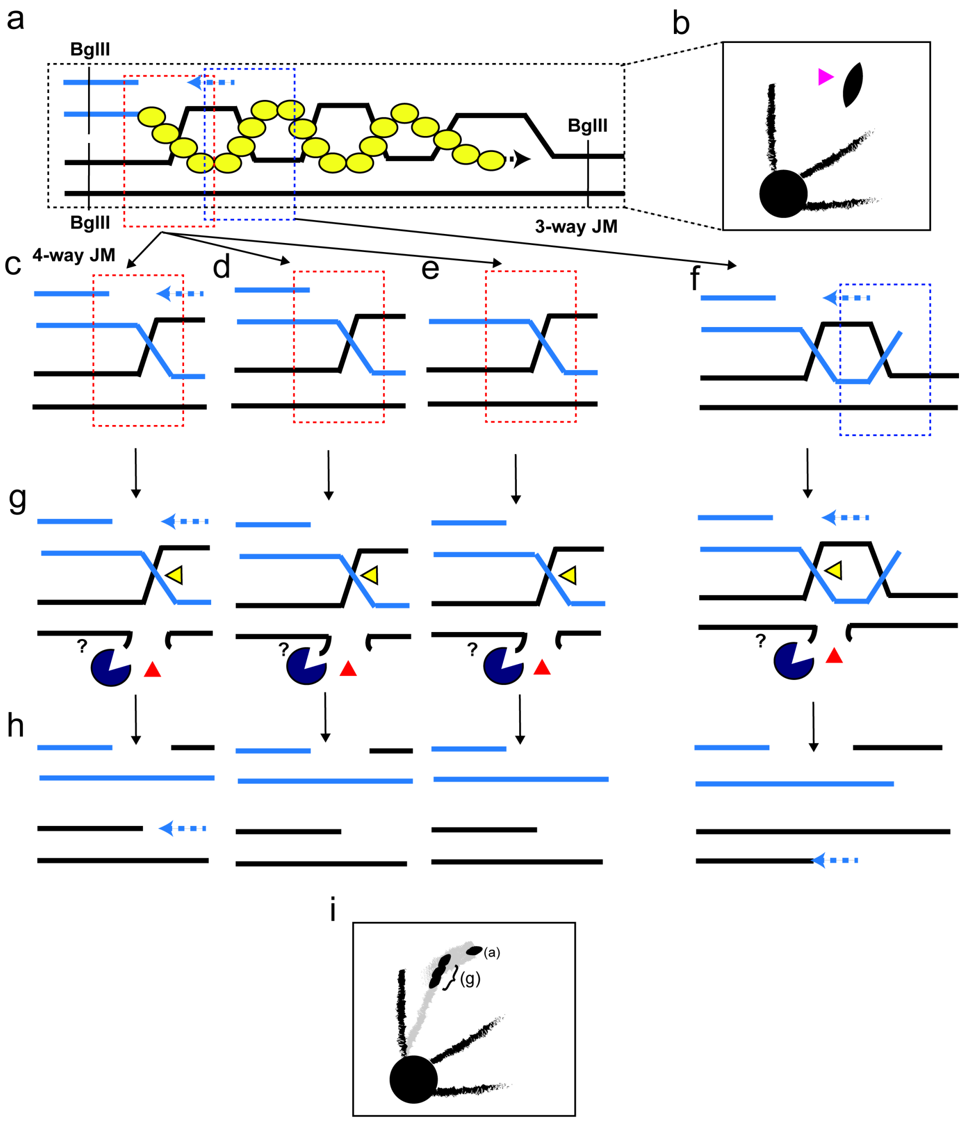

Supplementary Figure 5. Model showing the conversion of the 'spike' intermediate into the 'rubble' intermediate. (a) Schematic representing the multi-invasion intermediate (black dotted rectangle) that appears on 2D gels as a spike (b). We propose that only the BglII sites located outside of this area (marked as BglII) can be accessed and cut by BglII, which leads to accumulation of high-molecular weight, highly branched intermediate that we call 'spike' (see pink arrow head in (b)). Multi-invasion intermediates include areas of 4-way junctions (red dotted rectangle), which can be further classified as junctions where all strands are dsDNA (c), one strand is single-stranded (d) and where two strands are partially or completely single-stranded (e). These intermediates can also include areas of 3-way junctions (dotted blue rectangle) (f). (g) Processing of 4-way and 3-way junctions by structure-specific endonucleases, Mus81 (red arrowhead) and Yen1 (yellow arrowhead), leads to formation of nicked-intermediates that could potentially be processed further by resection (blue pacman). (h) Processing and also isomerization of the nicked-intermediate lead to varying levels of complexity and possibly reduced molecular weight. The schematic depicts the resolution of just one of the 4-way or 3-way junction in the multi-invasion intermediate in the structure. At a given point these structures will be heterogeneous and their specific position along the trajectory will depend on the amount of ssDNA and complexity of the intermediates (i).

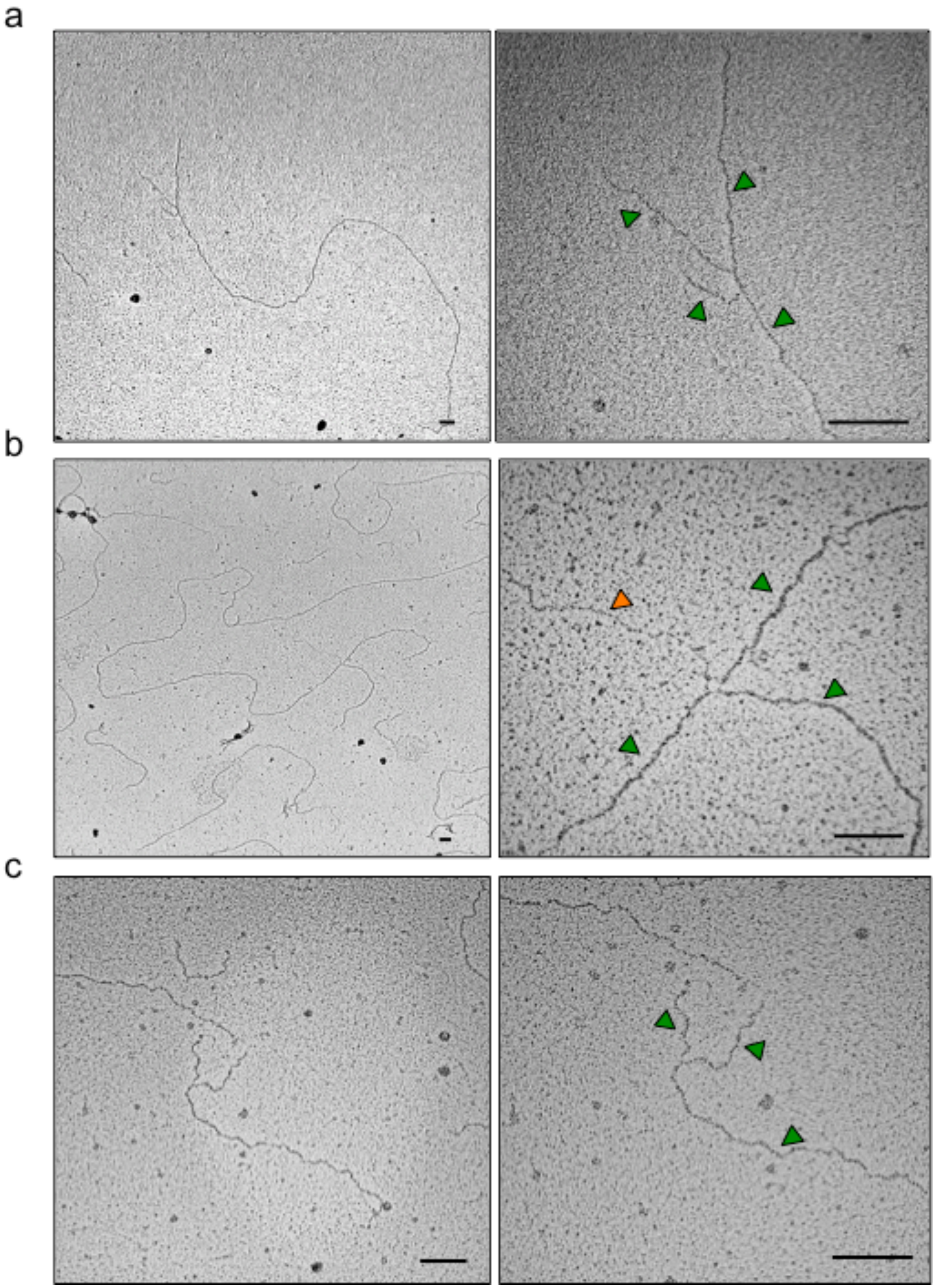

Supplementary Figure 6. Representative EM images showing joint molecules contributing to the rubble structure in *srs2Δ*. (a, b) 4-way junctions corresponding to the schematics drawn in Supplementary Fig. 5 (c) and (d) respectively. (c) 3- way junctions corresponding to the schematics drawn in Supplementary Fig. 5 (f). Green arrowheads denote double-stranded DNA and orange arrowheads depict single-stranded DNA. Entire field images including 3-way

junctions are shown (left) and enlarged views are shown (right). Note that rubble intermediates preserved in *srs2Δ* still include some 4-way and 3-way intermediates since the processing of these intermediates is incomplete. Scale bars correspond to 100nm.

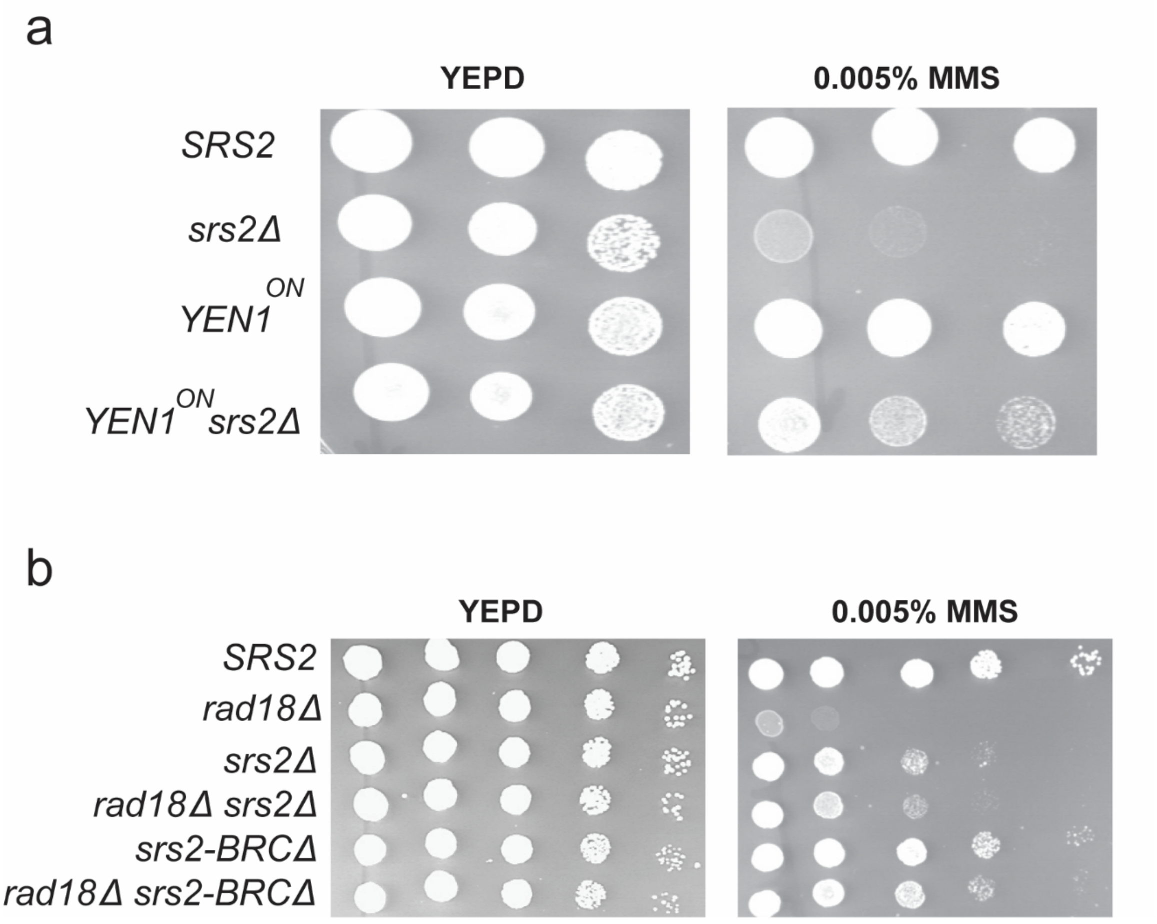

Supplementary Figure 7. MMS sensitivity of (a) *SRS2*, *srs2Δ, YEN1^{ON}*, and *YEN1^{ON} srs2Δ* strains. The MMS sensitivity was examined by plating of serially diluted cell cultures on YEPD and YEPD containing 0.005% MMS and (b) of *rad18Δ* in combination with *srs2-BRCΔ* and *srs2Δ* mutants.

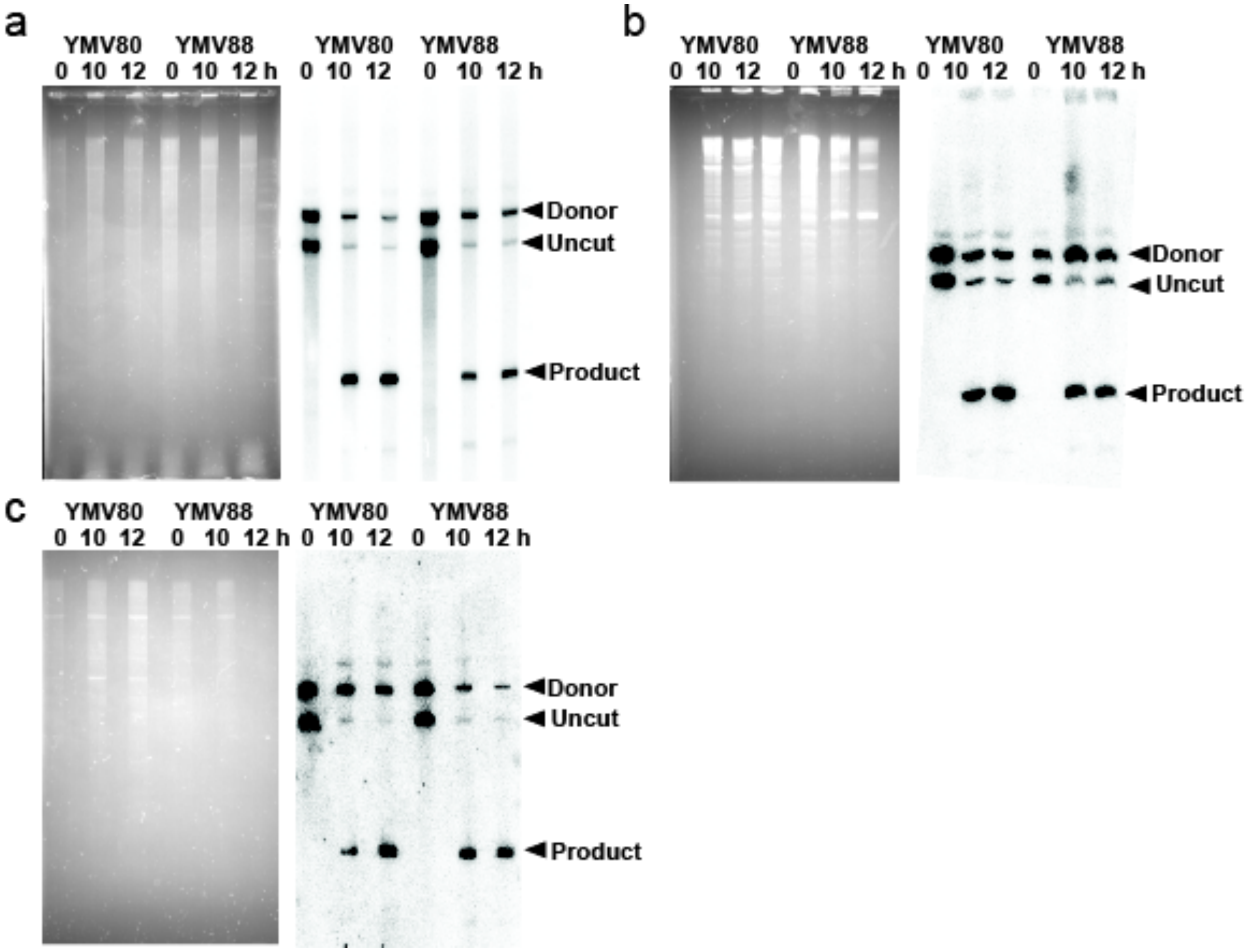

Supplementary Figure 8. Assaying DSB repair by SSA/BIR in YMV80 and YMV88 (a) Analysis of DSB repair following Acc65I digestion of genomic DNA and native gel electrophoresis as seen on ethidium bromide gels (left) following Southern analysis with hybridization to *LEU2*-specific probe (right). The location of Acc651 is the same as location of KpnI in [41]. (b) Analysis of DSB repair following in-plug digestion of chromosomal DNA with Acc65I as seen on EtBr gels (left) and following hybridization using *LEU2* probe (right). (c) Extraction of chromosomal DNA from agarose plugs by beta-agarase enzyme followed by Acc65I digestion as seen on EtBr gels (left) and following hybridization using *LEU2* probe (right).

Supplementary Table 1: Strain List

Strain Name	Genotype	Reference
AM3110	*hmlΔ::ADE1/hmlΔ::ADE3 MAT**a**-LEU2-tel/MATα-inc hmrΔ::HPH FS2Δ::NAT/FS2 leu2/leu2-3,112 thr4 ura3-52 ade3::GAL::HO ade1 met13 trp1::p304-BrdU snt1::(TEF1/BSD)$_5$*	This study
AM3257	AM3110, *but srs2::KANMX*	This study
AM3350	AM3110, *but srs2-K41A*	This study
AM3601	AM3110, but *srs2-BRCΔ*	This study
AM3332	AM3110, but *mus81:: ble^r*	This study
AM3610	AM3332, but *srs2::KANMX*	This study
AM3312	AM3110, but *yen1::HPHMX*	This study
AM3322	AM3312, but *srs2::KANMX*	This study
AM3451	AM3312, but *mus81::ble^r*	This study
AM3572	AM3110, but *YEN1^{ON}*	This study
AM3654	AM3572, but *srs2::KANMX*	This study
AM3658	AM3654, but *mus81:: ble^r*	This study
AM3391	AM3257, but *Mat**α**-inc-LEU2-tel*	This study
AM3457	AM3110, but *rad55:: ble^r*	This study
AM3462	AM3457, but *srs2::KANMX*	This study
AM3485	AM3350, but *rad55::ble^r*	This study
AM3489	AM3601, but *rad55::ble^r*	This study
AM3340	AM3110, but *rfa1-t33*	This study
AM3362	AM3340, but *srs2::KANMX*	This study
AM3768	AM3110, but *siz1::KANMX*	This study
AM3864	AM3601, but *siz1::KANMX*	This study

AM1291	AM1003, but *lys2::insA*$_4$ reporter inserted at 16kb position	[34]
AM3205	AM1291, but *srs2:: KANMX*	This study
AM1482	AM1003, but *lys2::insA*$_4$ reporter inserted at 36kb position	[34]
AM3206	AM1482 but, *srs2:: KANMX*	This study
AM2944	AM1003, but *ura3-29* reporter inserted at 36kb in Orientation 1	[10]
AM3201	AM2944, but *srs2::ble*^{r}	This study
AM2951	AM1003, but *ura3-29* reporter inserted at 36kb in Orientation 2	[10]
AM3200	AM2951, but *srs2:: ble*^{r}	This study
AM4064	AM3110, but *rad18::HPHMX*	This study
AM4067	AM3257, but *rad18::HPHMX*	This study
AM4068	AM3601, but *rad18::HPHMX*	This study
tGI354	*hmlΔ::ADE1 MAT**a**-inc hmrΔ::ADE1 ade1 leu2-3,112 lys5 trp1::hisG ura3-52 ade3::GAL::HO arg5,6::GAL::MAT**a***	[28]
tGI383	tGI354, but *srs2::LEU2*	[28]
tGI572	tGI354, but *rad55::LEU2*	This study
yDS178	tGI354, but *srs2::TRP1, rad55::LEU2*	This study
yAP47	tGI354, but *siz1::KanMX*	This study
yAP53	tGI383, but *siz1::KanMX*	This study
yGI96	tGI354, but *pol30-K127R::KanMX*	This study
yGI99	tGI354, but *pol30-K164R::KanMX*	This study
yGI102	yGI96, but *K164R::KanMX*	This study
AM3918	tGI354, but *srs2-BRCΔ*	This study
AM3967	AM3918, but *siz1::KanMX*	This study
YMV80	*hmlΔ::ADE1 MAT**a**Δ::hisG hmrΔ::ADE1 leu2-cs ade3::GAL::HO ade1 lys5 ura3-52*	[28]

YMV88	YMV80, but *srs2::KANMX*	[28]
AM3724	YMV80, but *srs2-BRCΔ*	This study

Supplementary Table 2: Analysis of BIR intermediates by electron microscopy

Strain	3-way JM[a]	4-way JM[a]	linear	Total
**No-DSB**	1	1	1401	1403
srs2Δ	68 **	32*	3760	3860
SRS2	34	13	3348	3395

[a] 3-way and 4-way junctions (joint molecules).

Supplementary Table 2. Analysis of BIR intermediates by electron microscopy. Table shows the number of 3-way junctions, 4-way junctions and of linear molecules that were analyzed during BIR in *SRS2* and *srs2Δ.* Strains lacking HO-endonuclease cut site (no-DSB) were arrested at G2 using nocodazole and were used as a control. ** and * indicate significant differences from *SRS2* with P-values of 0.0066 and 0.0379, respectively.

Supplementary Table 3: Primer List

Oligo Name	Description	5' – 3' sequence
OL550	FP: To amplify *srs2::KANMX* for deletion of *SRS2*	GTGATCGCTGACTCTGGTATTGGG
OL551	RP: To amplify *srs2::KANMX* for deletion of *SRS2*	CCGACGGATGTTGATGACCCATTG
OL552	FP: To confirm deletion of *SRS2*	GCTAGTCAAGAGCGATCCCCACTT
OL553	RP: To confirm deletion of *SRS2*	CCTCGAATTTCATTGACTTGGATG
OL2684	FP: To amplify *mus81:: ble*^{r} for deletion of *MUS81*	GCCCTTTAAGAGTGGCATCAACATTGGCGTAAACAAAGTTTCAAAGGATTGATACGAACACACATTCCTAGCATGAAAGCgacatggaggcccagaatac
OL2685	RP: To amplify *mus81:: Ble*^{r} for deletion of *MUS81*	ATATATGTATATATTAGTTAAAAGAATATCATCACTTTTTTCTTTATAAAACCTTGCAGGGATGACTATATTTCAAATTGcagtatagcgaccagcattc
OL236	FP: To confirm deletion of *MUS81*	GTTCTTGCTGCTCGTAAGAGACAC
OL537	RP: To confirm deletion of *MUS81*	CATATGCTTCTGCGATGGGCAGCG
OL1504	FP: To amplify *yen1::HPHMX* for deletion of *YEN1*	GGTAGTATATTGGCATTGAACACTGG
OL1505	RP: To amplify *yen1::HPHMX* for deletion of *YEN1*	CCTACTTTACAATCCACTATACGGTC
OL1506	FP: To confirm deletion of *YEN1*	GGCTATCCTTTTTAGACTCTTCTCC

OL1507	RP: To confirm deletion of *YEN1*	AATTCCCATATTTGTGAGACACCC
OL2751	FP: To amplify *rad55::Ble*^{r} for deletion of *RAD55*	CGCTATCAAAGATGTCAAGTACATTAAGGAAAGCTTTTAGATAAGGAAAAGAACTTATTAATAATATATAATATGAAAAT gacatggaggcccagaatac
OL2752	RP: To amplify *rad55::Ble*^{r} for deletion of *RAD55*	TTTCTTTGTAGTTGTTGGGTGGAATGTTGTCGTTTTTCCATTTTTTATTGTTTTCGGTTTTCGGTTTTTTATTTTACTAcagtatagcgaccagcattc
OL2753	FP: To confirm deletion of *RAD55*	GCGGAGGTCCCCTAAGTTCTTCAC
OL2754	RP: To confirm deletion of *RAD55*	TCGCCGCGTTGCAAGTACATTGTG
OL2690	FP: To create *srs2-BRCΔ* by insertion of pCORE for *delitto perfetto*	CATCCAAAATCAATGGCAATTACGCTCCTAAAAGTAGAGTTAAAAGTCCAGAAAAAAGGTACGCTCCAGAAACTACATCAgagctcgttttcgacactgg
OL2691	RP: To create *srs2-BRCΔ* by insertion of pCORE for *delitto perfetto*	AATGAAACTCCTGCCTACTAGGTACATTAGTTGTTGAAACGTATTGAGGTGCATACACCTTTTTCTTTGTAGGAGAATGGtccttaccattaagttgatc
OL2709	FP: To remove pCORE to create *Srs2-BRCΔ*	TCGAAACGCAAAACTCTATATTCCCACAGAAGAAACTTATTGAAAAATCAGGAGTTTCATTCTTCTACTGGGAAAAATATTCCTTTTCTGAGAAGAGAAG
OL2710	RP: To remove pCORE to create *Srs2-BRCΔ*	CTTCTCTTCTCAGAAAAGGAATATTTTTCCCAGTAGAAGAATGAAACTCCTGATTTTTCAATAAGTTTCTTCTGTGGGAATATAGAGTTTTGCGTTTCGA
OL3264	FP: To delete *rad18::HPHMX*	ATGGACCACCAAATAACCACTGCAAGCGACTTCACGACTACTTCAATACCGAGCCTGTACCAATTGGATACACTTTT

		GAcataggccactagtggatctg
OL3265	RP: To delete *rad18::HPHMX*	TTAATTGTTACCGGGTGGGTCTTTACTATATTCATTCAAGTCCATTAATTCTCTTGATAAGTCAGCATCAGTTGAATCTTagctgaagcttcgtacgc
OL3266	FP: To confirm deletion of *RAD18*	CCCGCTCTTCTATGGTATAATCAA
OL3268	RP: To confirm deletion of *RAD18*	CCAGGAACTCACTTCTCAGCAAGG
OL2641	FP: To amplify *siz1::KANMX* to delete *SIZ1*	CCATTTTAGAGCGAGCAGAAGGTT
OL2642	RP: To amplify *siz1::KANMX* to delete *SIZ1*	GAATAAGGTTGGTGTCGGAATACC
OL2643	FP: To confirm deletion of *SIZ1*	AAATCATCAACCTTGAGGCTAGGA
OL2644	RP: To confirm deletion of *SIZ1*	AAAACTGGCGTTTACTCCTGGTCA
